# Leadership and management in quality radiology

**DOI:** 10.2349/biij.3.3.e21

**Published:** 2007-07-01

**Authors:** LS Lau

**Affiliations:** International Radiology Quality Network, Canterbury, Victoria, Australia

**Keywords:** Quality and safety, quality systems, accreditation, medical imaging, interventional radiology

## Abstract

The practice of medical imaging and interventional radiology are undergoing rapid change in recent years due to technological advances, workload escalation, workforce shortage, globalisation, corporatisation, commercialisation and commoditisation of healthcare. These professional and economical changes are challenging the established norm but may bring new opportunities. There is an increasing awareness of and interest in the quality of care and patient safety in medical imaging and interventional radiology. Among the professional organisations, a range of quality systems are available to address individual, facility and system needs. To manage the limited resources successfully, radiologists and professional organisations must be leaders and champion for the cause of quality care and patient safety. Close collaboration with other stakeholders towards the development and management of proactive, long-term, system-based strategies and infrastructures will underpin a sustainable future in quality radiology. The International Radiology Quality Network can play a useful facilitating role in this worthwhile but challenging endeavour.

## A CHANGING ENVIRONMENT

Medical imaging and interventional radiology have been undergoing rapid advances in recent years. Patients now enjoy the benefits of earlier diagnosis and less invasive treatment alternatives with lower morbidity and mortality. The volume and complexity of work are steadily increasing but the supply of the professional workforce is not growing sufficiently to meet this increasing demand. From this perspective, modern radiologists are the victims of their success. This workload/workforce imbalance is one of the factors, which could potentially threaten the quality of care and patient safety.

The workplace environment and arrangements are changing. There are technological advances in diagnostic and interventional techniques. Picture Archive and Communication Systems (PACS) are becoming more available. These changes in infrastructure together with faster internet communication and more secure Virtual Private Networks are driving new service delivery models by applying clinical teleradiology.

Globalisation of healthcare, progressive corporatisation of radiology providers and threatening commoditisation of radiology services are emerging [1,2]. International clinical teleradiology is at the leading edge of this global healthcare model. Policy regulators and other healthcare providers are monitoring this evolving model with keen interest. Commercialisation and corporate ownership of radiology practices by large listed companies are taking place in some communities. To meet budget expectations from the shareholders [3], pressure is mounting on radiologists to do more for less. Skyrocketing healthcare costs lead to outsourcing of services [4], which is not limited to medical imaging and interventional radiology. Some observers comment on the maturing commoditisation of international clinical teleradiology, treating the profession like commodities such as cotton or sugar, which could be traded with futures contracts [5].

In some countries, there is an increasing number of radiologists placing greater emphasis on lifestyle, electing to balance work with family commitments and opting out of after hours call duties if possible, despite the increasing demand in 24-hour services.

Further convergence of clinical radiology and medicine has created battlefields of new turf. For example, cardiac imaging joins vascular intervention and ultrasound as another front where clinicians and radiologists jostle for control. Workforce shortages that are not meeting the increase in service demands is a major dilemma confronting radiologists when considering turf debates.

These changes are evolving and will, no doubt in some way, impact on the quality of service delivery. Radiologists and professional organisations must provide leadership, manage these challenging conditions effectively and ensure that the quality of care and patient safety are not compromised as a result of these major changes.

## QUALITY MEDICAL IMAGING AND INTERVENTIONAL RADIOLOGY

### The ideal

Quality in medical imaging and interventional radiology may be defined in many ways and from different angles. One of these is: ‘A timely access to and delivery of integrated and appropriate radiological studies and interventions in a safe and responsive facility and prompt delivery of accurately interpreted reports by capable personnel in an efficient, effective and sustainable manner.’

The above statement captures the desirable performance parameters of the National Health Performance Framework [6], i.e.:

1. Access: the ability of a patient to obtain medical imaging and interventional radiology at the right place and right time irrespective of income, physical location and cultural background;
2. Integrated: the ability to provide uninterrupted and coordinated care across facilities and practitioners. In medical imaging and interventional radiology, the availability of and access to relevant clinical history, indications and findings of previous radiological studies or interventions, and the opportunity to discuss with the referring physician or patient are essential components, which can significantly influence the diagnostic study, intervention selection, interpretation and follow-up management options;
3. Appropriate: the care, intervention or action provided is relevant to a patient’s need and is based on established standards. The radiologist is the consultant assisting the referring physician and patient in selecting the most appropriate radiological study or intervention for the clinical condition, based on evidence-based practice guidelines;
4. Safe: the avoidance or minimisation of actual or potential harm from medical imaging or interventional radiology, including radiation exposure, magnetic fields, contrast media etc.;
5. Responsive: the primacy of a patient is recognised and respected. The facility is patient-oriented and practices these aspects: respect for patient’s dignity and confidentiality, participation in choices or decision-making, prompt, and good quality of amenities and choice of provider;
6. Timely report and accurate interpretation: the medical imaging report should be accurately interpreted and the interventional procedure precisely documented and delivered to the referring physician in a timely manner for optimal patient management. Reliable means of report delivery and confirmatory mechanisms are essential especially in the case of urgent or unexpected findings;
7. Capable: the facility’s and individual’s capacity to provide medical imaging and interventional radiology based on skill and knowledge;
8. Efficient: achievement of the desired results with the most cost-effective use of resources;
9. Effective: the care, intervention or action should be effective in achieving the desired outcome;
10. Sustainable: the system must be capable in providing infrastructure such as workforce, facilities and equipment, and be innovative and responsive to emerging needs.

### The reality

In practice, the reality could be a departure from the above ideal parameters. There are potential threats to quality and safety due to workplace, workload, workforce and budget challenges. These examples include inadequate capital funding for the replacement of rapidly outdated equipment in the workplace, escalating workload with increasing complexity, recruitment and retention of radiology professionals due to a global shortage, efficiency and productivity expectations from facility managers, and the shrinking budget that is not keeping up with inflation.

### The performance bar

Radiologists must lead and convince facility managers and lay administrators that the quality, workload and performance metrics, i.e., access waiting list, workload, accuracy, turn-around time, quality and safety issues, etc. are interlinked [7]. At any given level of workforce, the staff output is, by and large, finite to maintain quality and safety. Radiologists must be the leading members of the decision-making team in relation to the allocation of resources and ensure that the mix is optimised and realistic (Figure 1a). Increased demands and/or expectations on the performance of one, e.g. workload, will, by definition, impact adversely on one or more of the remaining deliverables with the same resources (Figure 1b). It is thus essential that decisions on resource allocation and performance expectation reflect this reality to minimize the facility’s risks. The output ‘pie’ is, after all, only so big! The challenge for the providers is to try and achieve the best and realistic outcome within the limited resources.

**Figure 1a:**
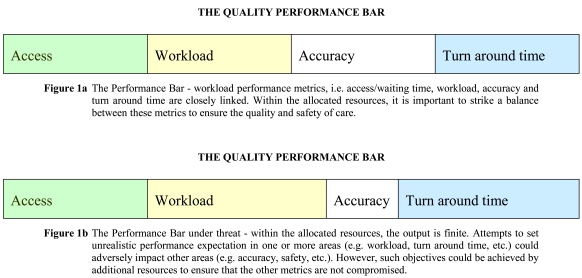
The Performance Bar - workload performance metrics, i.e. access/waiting time, workload, accuracy and turn around time are closely linked. Within the allocated resources, it is important to strike a balance between these metrics to ensure the quality and safety of care.

**Figure 1b:**
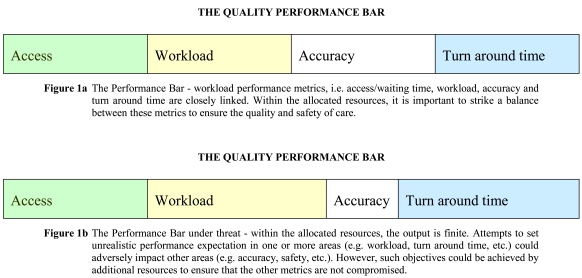
The Performance Bar under threat - within the allocated resources, the output is finite. Attempts to set unrealistic performance expectation in one or more areas (e.g. workload, turn around time, etc.) could adversely impact other areas (e.g. accuracy, safety, etc.). However, such objectives could be achieved by additional resources to ensure that the other metrics are not compromised.

## QUALITY LEADERSHIP AND MANAGEMENT IN MEDICAL IMAGING AND INTERVENTIONAL RADIOLOGY

To survive and be successful under the changing environment and to ensure quality and safety outcomes, leadership from the profession and collaboration with other stakeholders to jointly develop and manage long term system-based strategies, are required. It is important to strike a balance between quality, safety, cost, sustainability, and clinical and patient outcome improvements.

### Radiologists as leaders

Radiologists are committed to the principles of professionalism and professional responsibilities. The professionalism principles include the primacy and autonomy of the patients and social justice [8]. Professional responsibilities cover scientific knowledge, professional competence, quality of care, access to services and just distribution of finite resources.

Consumers expect professional leadership by self-regulation in the first instance, i.e. by addressing workforce training and professional development issues, and by setting quality standards and developing service delivery models, which are in the consumers’ best interests [9]. Radiologists must be the leaders for the promotion of quality, and consumers’ advocates for quality improvement, appropriate and sustainable use of medical imaging, and interventional radiology.

In addition to professionalism, there are other reasons for radiologists to be leaders for quality. For example, quality is acknowledged as a marketing differentiator in an increasingly competitive environment. Malpractice insurers recognize the link between quality and risk management. In fact, some insurers offer premium reductions to individuals or practices participating in quality improvement activities.

### Governments and payers as leaders

Some health policy regulators and payers use quality and evidence-based radiology as levers to manage the increasing demand for services. They expect value, better clinical and economic outcomes, and work towards ultimate system sustainability. A few independent organisations lead and promote quality by rewarding providers with higher re-imbursement for outstanding quality services (pay-for-performance).

Governments, politicians and bureaucrats must demonstrate their commitment to quality and lead by working with other stakeholders to develop and manage sustainable re-imbursement models, to reward quality providers and to invest in research and development on quality and safety infrastructure. Government funding on system-based quality and safety R&D is minuscule when compared to other industries with a similar budget. When there is a significant change in the practice delivery model (e.g. telemedicine), law makers must lead by providing the necessary regulatory or legal framework to ensure that quality is not compromised and the delivery model is in the consumers’ best interest.

### Other stakeholders as leaders

The consumers of medical imaging and interventional radiology include patients and referring physicians. There is an increasing awareness of and expectation for quality services by consumers. Consumers can lead the quality push by: becoming better informed, providing feedback, acknowledging the community’s limited resources, recognising their social responsibilities and requesting services appropriately.

## STAKEHOLDERS’ COLLABORATION AND QUALITY SYSTEMS

Cooperation and collaboration between the stakeholders are synergistic and will add value in spearheading the push for quality. Collaboration is strength! Collaboration is needed among all stakeholders (i.e. consumers, providers, payers, etc.), professional organisations (i.e. local, national, international, etc.), professional groups (i.e. radiologists, technologists, physicists, etc.) and disciplines (i.e. radiologists, other clinical disciplines, etc.). Collaboration will breakdown barriers, identify common goals and pave the way towards better quality outcome for patients.

Radiologists are initiators, facilitators and participants. Over a long time, radiologists have led, developed and successfully managed a range of quality programs and processes, while addressing individuals’, practices’, national and international needs. Quality systems are recognised as effective risk control measures. It is important for radiologists and professional organisations to lead, develop, maintain, manage and improve such quality systems. Well-directed team work is equally, if not more, important than individuals in delivering systematic improvement.

### Quality systems for radiologists

There are jurisdictional and institutional requirements addressing the quality standards for radiologists. These requirements include the qualification and certification needed following the completion of a training program covering theoretical knowledge and practical experience. There is a progressive trend by authorities and professional organisations requiring radiologists to demonstrate the on-going proof of practice competency by re-certification or re-validation. This may be via examination or participation in a Continuing Medical Education (CME) or Continuing Professional Development (CPD) Program. Professional organisations usually provide and manage the infrastructure or systems necessary to support these requirements. Credentialing and privilege of practice is granted by institutions to radiologists following confirmation of training, experience, insurance cover and participation in on-going learning.

### Quality systems for facilities

Quality efforts for radiology facilities range from participation in facility-based quality improvement measures by applying quality maps, measurable metrics, performance indicators and audits or formal radiology-specific accreditation programs dealing with quality issues in a more comprehensive and systematic way [10, 11]. Radiologists can lead and manage facility-based quality infrastructure by instituting quality improvement measures, developing metrics which are easily measurable, implementing changes which are readily achievable and leading the facility’s participation in a formal accreditation program. These collective efforts will minimize the facility’s risks and benefit the consumers.

National radiology-specific accreditation programs for facilities are available in Australia from the Royal Australian and New Zealand College of Radiologists (RANZCR) and National Association of Testing Authorities [12]; in Finland, from the Radiation and Nuclear Safety Authority; in Korea, from the Korean Institute of Accreditation in Medical Imaging; in New Zealand, from the International Accreditation New Zealand and in the United States, from the American College of Radiology (ACR), American Institute of Ultrasound in Medicine and the Inter-Societal Commission for the Accreditation of Vascular Laboratories. Generic quality management accreditation is available through the International Organization of Standardization (ISO) agencies.

### National quality systems

As a demonstration of their leadership and commitment to quality in medical imaging and interventional radiology, radiologists and professional organisations around the world have led, collaborated, developed and contributed to a range of measures aiming to secure a sustainable, quality future within the finite resources. These include an education campaign for the stakeholders to promote appropriate utilisation, e.g. the publications of Appropriateness Criteria (ACR); Clinical Referral Guidelines (Hong Kong College of Radiologists); Imaging Guidelines (RANZCR) and Making the Best Use of a Department of Clinical Radiology [Royal College of Radiologists (RCR) and European Association of Radiology (EAR)].

Other national system-wide quality improvement initiatives include: the Continuous Improvement in Radiology Information System (CIRIS) in the United Kingdom; the Medical Excellence in Diagnostic Imaging Campaign (MEDIC) in the United States and the Quality Use of Diagnostic Imaging (QUDI) Program in Australia.

CIRIS [13] was developed in partnership between the Royal College of Radiologists (RCR) and the College of Radiographers, with financial support and advice from the Department of Health and the Scottish Executive. It is available to the NHS Trust to: bring the stakeholders together; provide online service to assist with the compliance to standards, regulatory requirements, governance and quality improvement; and ensure that patients receive the best care possible. In addition to record keeping, compliance and quality improvement, benchmarking is a useful feature. An individual department can benchmark its waiting time or the age of a piece of equipment across the country. Such information may support the business case for more resources or equipment update.

MEDIC [14] was developed by the ACR to educate the public, media, physicians and government officials that by establishing quality and safety standards for medical imaging providers and facilities, Medicare and American taxpayers can save billions of dollars while improving quality of care; provide a repository of information, government reports and peer-reviewed studies illustrating how inappropriate imaging lowers quality of care and how the cost associated with unnecessary tests threatens the solvency of Medicare/Medicaid and drains the healthcare system of billions of dollars annually; explain the important role that radiologists have in providing quality patient care; and how the public can help protect quality care for the nation’s seniors while lowering healthcare costs.

The QUDI Program [15, 16] was initiated by RANZCR and funded by the Commonwealth Department of Health and Ageing. It is a AUD$5 million program over 5 years. The vision is a comprehensive, long-term strategic approach to promote sustainable, evidence-based, appropriate and quality use of medical imaging, focusing on and addressing the needs of the key stakeholders including consumers, referring physicians, providers and payers. The integrated projects are designed so that they will complement each other and add value to each sub-program. A built-in program evaluation is a key feature of QUDI. It is envisaged that when these projects are completed and the findings implemented, it will lead to a significant improvement of medical imaging services in Australia.

There are common features associated with these national quality initiatives. They are usually developed by the profession as it is committed to the professionalism principles, aiming to deliver quality and safety of care within finite resources and to work towards system sustainability. This requires leadership, vision and dedication. These initiatives aim to develop long-term, pro-active, system-based, multi-tiered, and multi-dimensional plans; to inform, engage, collaborate and seek stakeholders’ support and to define roles and clarify responsibilities for all stakeholders.

The biggest challenge for such initiatives is failure to deliver on what was intended due to a variety of reasons. Financial impact to radiology facilities and political considerations might bring adverse effects and threaten support. Individual workload and organisational resources might be inadequate. Inevitable change of leadership and key personnel could be other possible risks threatening these long-term plans.

### International quality efforts

International efforts in promoting quality in radiology include the works of the Asian Oceanian Society in Radiology, European Association of Radiology, Inter-American College of Radiology, International Atomic Energy Agency, International Commission on Radiological Protection, International Radiology Quality Network, International Society of Radiographers and Radiation Technologists, International Society of Radiology, Radiological Society of North America, the World Health Organization, etc.

Each organisation’s quality focus depends on its objectives, the needs of its members and the areas of interest. Given a diversified range of quality efforts by these professional organisations and finite resources, the profession’s aim should be to add value and not to re-invent the wheel. This can be achieved by good communication and mutual sharing of information, resources and feedback between projects or programs, and within and between organisations. The profession should work towards collective and integrated efforts at all levels and among all team members and organisations.

## THE INTERNATIONAL RADIOLOGY QUALITY NETWORK (IRQN)

The IRQN was founded in 2002. It is a network of organisations. The current members are the American College of Radiology (ACR), Asian and Oceanian Society in Radiology (AOSR), European Association of Radiology (EAR), now known as European Society of Radiology (ESR), Inter-American College of Radiology (CIR), International Society of Radiographers and Radiological Technologists (ISRRT), International Society of Radiology (ISR), Japan Radiological Society (JRS), Radiological Society of North America (RSNA), Royal College of Radiologists (RCR), Royal Australian and New Zealand College of Radiologists (RANZCR) and Global Steering Group in Diagnostic Imaging and Laboratory of the World Health Organization (WHO). The network’s objectives are to promote quality in radiology through collaboration, experience sharing and mutual assistance [17, 18].

The IRQN is poor in financial resources but rich in professional leadership assets. It is supported by a wealth of experienced people and organisations, which it can readily consult for expert opinion and assistance. It regards itself as one of the leaders to champion, facilitate, develop and manage an infrastructure towards a sustainable quality future, in collaboration with other stakeholders at an international level. Its quality efforts include:

- Undertaking a quality awareness program by participating in quality sessions in international conferences including the ACR, AOSR, ESR, International Atomic Energy Agency (IAEA), ISR and RSNA;
- Hosting a Quality Improvement in Radiology Conference in collaboration with the RANZCR in 2003;
- Publishing its quality activities in radiology journals;
- Developing and harmonising Principles for International Clinical Teleradiology;
- Implementing a “Quality Improvement in Practices” paper competition in collaboration with the Journal of the American College of Radiology. The aim is to promote awareness either by active research and manuscript contribution or by passive learning through reading and applying the published quality improvement techniques; and
- Establishing a Performance Metrics/Quality Indicator Workgroup to develop metrics benchmarking. This will commence with a pilot project by initially developing and defining an indicator and piloting data collection. These steps will help to identify the issues involved with a voluntary multi-national, multi-facility undertaking prior to the development of a more comprehensive benchmarking project internationally.

In advancing the quality agenda, there are potential collaborations and synergies between the IRQN and other related organisations. For example, other organisations may be informed of IRQN developments, be supportive of the network’s principle, objectives and quality initiatives, and relay this information to their members. Organisations can avoid duplication of efforts by improving link and communication. Collectively, the network and other organisations can share quality resources, provide networking opportunities, co-sponsor quality segment in conferences and jointly approach governments for the funding of quality initiatives.

## DISCUSSION

The radiology working environment is rapidly changing due to globalisation of healthcare, corporatisation of radiology facilities, commercialisation of teleradiology and possible commoditisation. Such changes will upset the existing equilibrium but may offer new opportunities. For example, there are many pros and cons associated with international clinical teleradiology [9].

The increase in demand due to an aging population and the skyrocketing of healthcare costs are becoming concerns in some countries, which if uncurbed will be unsustainable. The payers are therefore keen to cap healthcare expenditure. The outsourcing of healthcare including medical imaging will grow, as a means of saving cost: the rate depends on implemented local standards and guidelines [19]. Such arrangements will challenge traditional arrangements and will be controversial.

In this environment, radiologists and the profession should not reject changes because they are upsetting the existing equilibrium but transform these concerns into opportunities. However, these fundamental changes in practice will require timely leadership and thoughtful development of new ethical, legal and quality framework by the profession and regulators. With awareness, commitment, leadership, collaboration, planning, good management and appropriate utilisation, it may be possible to achieve both quality and economic objectives.

Radiologists and the profession must uphold their duty of care and ensure that quality and safety are not compromised as a result of change in practice and budgetary pressures. They will lead and collaborate with other stakeholders to develop a long-term, integrated, proactive and system-based framework rather than reacting to short-term issues. The profession must be the prime movers, leaders and facilitators with collaboration and support from all other stakeholders. The challenge for the leaders is to develop plans that will bring better outcome to ALL stakeholders and be sustainable for the long term. Quality leadership in medical imaging and interventional radiology is a marathon and requires patience and perseverance.

Providers and payers when leading the quality agenda should recognise the difference among quality control, quality assurance and quality improvement [20]. Using chest X-ray as an example, quality control is the rejection and re-doing of a poorly exposed or positioned film to ensure that the final view is diagnostic and meet the minimal referrer expectation. Quality assurance requires a little more effort, i.e. well- documented procedure manuals, exposure charts, processor quality control measures, staff training, etc., to reduce the percentage of poorly exposed or positioned films. Quality improvement is a proactive process, by analysing, developing and implementing ongoing improvement measures for each and every step of the examination so that the final film is better exposed, positioned and diagnostic with minimal radiation.

The importance and benefits of a system-based approach to the promotion of quality should be emphasised. In an ideal world, it would be good to have A systems supported by A teams. However, in reality and with limited resources, it is far better to have A systems supporting B teams rather than the reverse. Good systems will guide the facilities to do the job right the first time and save time and cost.

A hurdle regularly faced by professional organisations in managing long-term strategies is the turnover of key personnel and office bearers, leading to inevitable loss of corporate memory and direction. However, the profession is optimistic that as a result of the dedication of the radiologists and the commitment of the professional organisations, it should be possible to maintain ongoing interest, leadership and direction.

Quality efforts are expensive in the short- and medium-term, especially if uncoordinated. However, they are inevitable and indispensable in the long term as an integral part of professionalism and risk minimisation. Professional leadership by radiologists and professional organisations via informing the uninformed and converting the sceptics is the only sustainable way forward. Closer collaboration between the profession, governments and other stakeholders will be a major step forward towards achieving cost-effective and appropriate use of medical imaging and interventional radiology, and better delivery of care in the long term. The International Radiology Quality Network can play a useful facilitating role in this worthwhile but challenging endeavour at an international level.
